# Elevated air quality index and fine particulate matter levels contribute to the poor prognosis and progression of nonsmall‐cell lung cancer: A cohort study combined with external validation

**DOI:** 10.1002/cam4.4701

**Published:** 2022-03-21

**Authors:** Jing Li, Xiaoying Wei, Ling Gu, Linya Qiu, Mengqi Xiang, Huachuan Zhang, Lei Xia, Wenying Pan, Zhenyu Yang, Xiaoli Zhou, Daxiong Zeng, Junhong Jiang

**Affiliations:** ^1^ Department of Medicine, Respiratory, Emergency and Intensive Care Medicine The Affiliated Dushu Lake Hospital of Soochow University Suzhou China; ^2^ Department of Medicine, Respiratory, Emergency and Intensive Care Medicine The First Affiliated Hospital of Soochow University Suzhou China; ^3^ Gusu District Center for Disease Control and Prevention Soochow People's Republic of China; ^4^ Department of Medical Oncology, Sichuan Cancer Hospital Medical School of University of Electronic Science and Technology of China Chengdu China; ^5^ Department of Thoracic Surgery, Sichuan Cancer Hospital Medical School of University of Electronic Science and Technology of China Chengdu China

**Keywords:** air quality index, NSCLC, PM2.5, prediction model, prognosis

## Abstract

**Background:**

Poor air quality can result in a variety of respiratory disorders. However, the air quality index (AQI) and the level of fine particulate matter (PM2.5) on the progression and prognosis of nonsmall‐cell lung cancer (NSCLC) are unclear.

**Methods:**

We launched a cohort study focused on the relationship between air quality and overall survival as well as progression, incorporating data from 590 patients with NSCLC in our medical center between November 1, 2013 and March 1, 2016. Forty‐nine patients from Sichuan Cancer Hospital were used for validation.

**Results:**

Cases with poorer AQI 6 months before NSCLC diagnosis were more likely to progress to stage III to IV NSCLC than controls (OR = 2.61, 95% CI 1.35–5.24, *p* = 0.005). Similarly, if exposed to high levels of PM2.5 during these 6 months, overall survival was poor (HR [95% CI] = 1.53 [1.13, 2.07], *p* = 0.006). According to multivariate analysis, age, gender, KPS, PM2.5, hyperlipemia, and NSCLC stage were independent risk factors of overall survival. A predictive model developed by these factors above yielded a favorable agreement (C‐index = 0.758) on the calibration curve. External validation was conducted by 46 patients from Sichuan Cancer Hospital displaying an AUC of 0.724 (0.684–0.763).

**Conclusions:**

PM2.5 and AQI levels affect disease progression and long‐term survival in NSCLC patients. An overall survival prediction model based on the PM2.5 level can help clinicians predict the risk of death in NSCLC.

## 1 INTRODUCTION

Air pollution, especially PM2.5, is a risk factor for many respiratory diseases.^1^ Elevated levels of PM2.5 can be found in air pollution environments worldwide. This can also be seen in many environments where smoking and cooking or heating through solid fuels is practiced..^2^, ^3^ PM2.5 can penetrate deep into the lungs and be deposited in the small airways,^4^, ^5^ whose physiology is vital for triggering respiratory diseases.^6^ Anatomically, the small airway has a small cross‐sectional area with an internal diameter of <2 mm.^7^ Small airways tract impairment contributes significantly to airflow obstruction^8^, ^9^ and airway inflammation,^10^, ^11^, ^12^ both risk factors for lung cancer. However, PM2.5 is related to increased oxidative stress and lung inflammation.^13^, ^14^

Lung cancer is a relatively common tumor and a significant cause of death from malignancy worldwide.^15^, ^16^ Chemotherapy for NSCLC is less effective than for SCLC, and thus approximately 50% of patients with NSCLC face cancer recurrence even if surgical resection is performed at early diagnosis.^17^ However, despite numerous clinical studies, 5‐year overall survival rates remain as low as 14%–17% for all stages of NSCLC^18^, ^19^ and even lower (6%) for SCLC.^20^ For the epidemiological studies and Virchow hypothesis, people prone to chronic inflammation are at elevated risk of NSCLC, and underlying inflammatory and infections responses are linked to nearly 20% of deaths worldwide.^21^ And air pollution has a catalytic effect on respiratory tract inflammation.^1^, ^22^ To our knowledge, few investigations have shown whether reduced PM2.5 can be related to long‐term prognosis as well as tumor progression in lung cancer.

To explore the correlation between air pollutants from different tumors and the progression and long‐term outcome of lung cancer patients, we launched a cohort study to obtain the air quality of patients for 6 months before their diagnosis. We correlated it with the survival status and disease progression of NSCLC patients to construct a survival prediction model. Data from Sichuan Cancer Hospital were also collected for external validation.

## 2 METHODS

### 2.1 Patients

**FIGURE 1:**
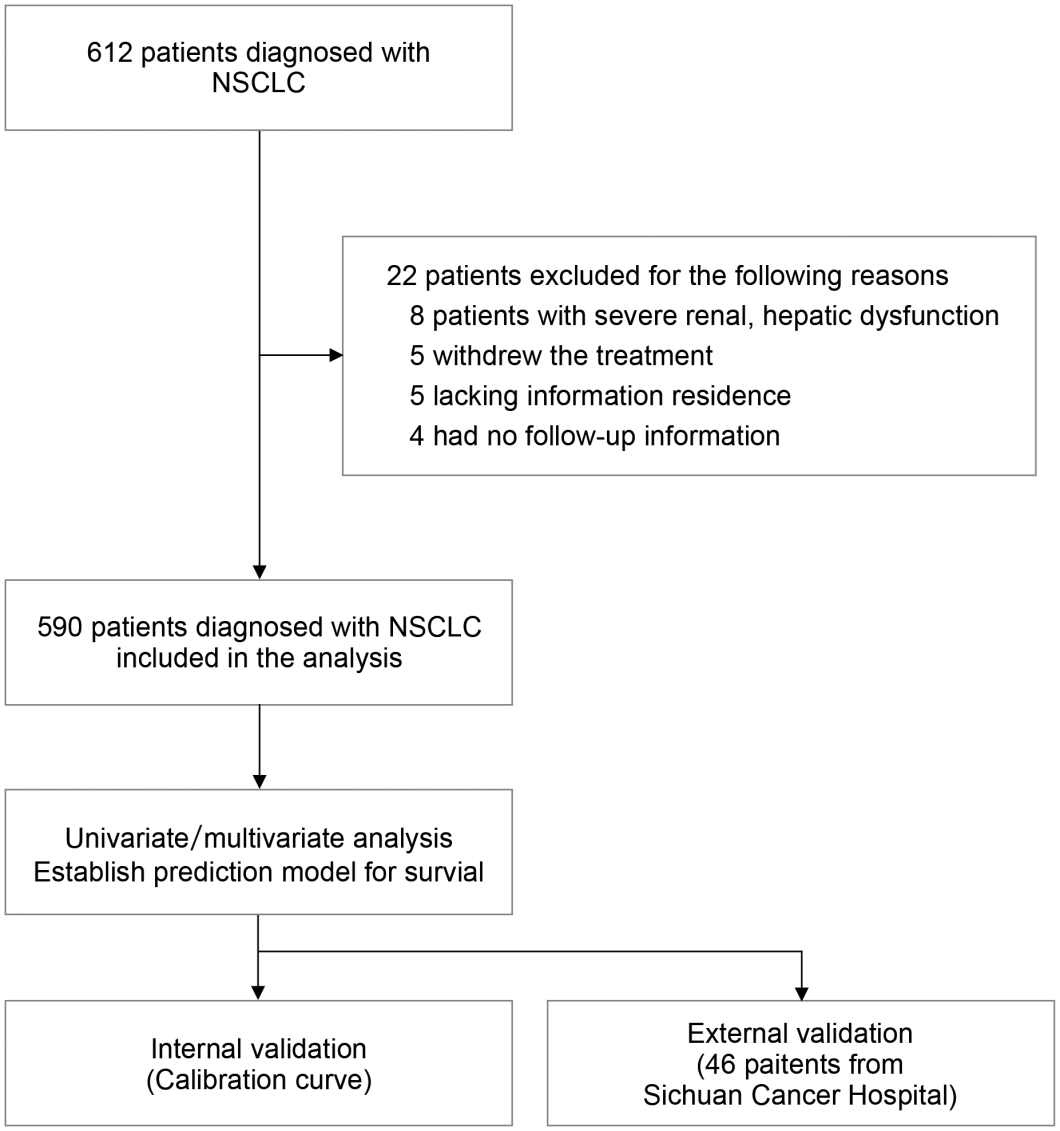
Flow chart for patients’ selection

We launched a cohort investigation that included data from 590 patients with NSCLC at our medical center between November 1, 2013 and March 1, 2016. The deadline for the follow‐up was December 1, 2021. The diagnosis of NSCLC was made concerning pathological diagnostic criteria. A flow chart of the enrollment process is displayed (Figure 1). Mortality data were obtained from the hospital registry, and the death time was confirmed by reviewing the electronic medical records or by phone follow‐up. Air quality‐related parameters were referenced to officially published data (http://www.cnemc.cn). The air quality assessment of patients for the 6 months prior to the onset of lung cancer was analyzed by calculating a 6‐month average for every patient. The median follow‐up period was 36.4 months. The database was performed by an independent researcher excluded from the patient care. Informed consent was achieved from their immediate family members or patients themselves. All research projects conformed to the guidelines of the Soochow University Ethics Committee and followed the Declaration of Helsinki. Data from 46 patients with NSCLC at Sichuan Cancer Hospital were applied for external validation.

### 2.2 Statistical analysis

NCSS‐PASS software version 12.0 was used for sample size calculation. Missing data were filled in using a machine‐learning model with the “mice” package in R. Continuous variables were displayed as mean ± standard deviation and median to the interval. The median was used as the cutoff value. The Mann–Whitney *U* test and unpaired *t* tests were applied for comparisons. Categorical variables were displayed as percentages or using the κ^2^ test. Cumulative mortality was displayed as Kaplan–Meier curves and analyzed by the log‐rank test. Univariate and multivariate survival analyses of overall survival (OS) were estimated by Cox regression models. The significance of variates on prognosis was analyzed visually by forest plots.

Variables with statistical significance in the multifactor analysis were used as predictor variables. The contribution of each factor was calculated and presented in the form of a nomogram plot with 1000 self‐directed validations. When using the Nomogram plot, locate the position of each variable on the axis, then draw a line on the integral axis, sum the scores of all variables, draw the line at the lower line of the Nomogram plot, and draw a line on the total integral axis to determine the events' probability. Calibration was used to assess the consistency of the models developed. The coherence of each model was visualized using scatter plots and analyzed using 1000 bootstrap methods. Statistical analyses were performed using the “ggplot2,” “rms,” “risk regression,” “surminer,” and “PredictABLE” packages of R software (version 4.0.5).

## 3 RESULTS

A total of 590 patients with NSCLC were enrolled in this investigation. The median age was 64 (58, 70). Among them, 407 (69.0%) cases were male, and 183 (31.0%) patients were female. The mean BMI level was 22.69 ± 3.15. There were 378 (64.0%) cases of adenocarcinoma and 187 (32.0%) cases of squamous carcinoma. Smokers accounted for 51.0% of the included population, with 301 cases. The median KPS score was 90. A total of 416 patients died, with a mortality rate of 71.0%, including 54% in stage I and 78.0% in stage IV, with a significant difference (*p* < 0.001). The overall median survival was 24.17 (9.9, 50.99) months. It was 38.9 (12.87, 59.8) for stage I patients and 20.4 (9.72, 48.59) for stage IV patients, again with statistical significance (*p* = 0.012).

In terms of air quality, 6 months before the patient's diagnosis, AQI, air quality classification, PM2.5, NO_2_, CO, PM10, and O_3_ all differed from the patient's NSCLC classification. Stage I patients with NSCLC were exposed to an AQI of 86 (81, 91) compared with 90.5 (84, 104) for stage IV patients, with a difference of statistical value (*p* < 0.001). Similarly, the ambient PM2.5 in Stage I NSCLC patients were 55 (47, 64) compared with 62 (53, 77) in Stage IV patients. PM10 was 72 (66, 87) versus 82 (70, 97) (*p* < 0.05). The median CO and O_3_ were 0.91 (0.82, 1.05) and 110 (57, 125.75), respectively.

For long‐term complications, among these 590 patients, there were 224 (38.0%) cases of hypertension, 57 (10.0%) cases of diabetes mellitus, 52 (9.0%) cases of hyperlipidemia, 11 (2.0%) cases of heart failure, and 15 (3.0%) cases of the acute coronary syndrome. Among them, hypertension and hyperlipidemia were statistically different in distribution among different NSCLC stages (Table 1).

**TABLE 1:** Study participant characteristics at enrollment

Variables	Total (*n* = 590)	Cohort, median (IQR)				*p* value
		Stage I (*n* = 94)	Stage II (*n* = 62)	Stage III (*n* = 121)	Stage IV (*n* = 313)	
Baseline data						
Age, Median (Q1, Q3)	64 (58, 70)	65 (59, 70)	64 (59, 68.5)	64 (57, 69)	63 (58, 70)	0.851
Gender, *n* (%)						0.797
Female	183 (31)	27 (29)	17 (27)	41 (34)	98 (31)	
Male	407 (69)	66 (71)	45 (73)	80 (66)	216 (69)	
BMI, Mean ± SD	22.69 ± 3.15	22.38 ± 3.3	22.62 ± 3.06	22.85 ± 3.15	22.73 ± 3.13	0.732
Pathological type, n (%)						0.485
Adenocarcinoma	378 (64)	57 (61)	36 (58)	77 (64)	208 (66)	
Mixed lung cancer	25 (4)	5 (5)	5 (8)	6 (5)	9 (3)	
Squamous carcinoma	187 (32)	31 (33)	21 (34)	38 (31)	97 (31)	
Smoking, n (%)						0.32
No	289 (49)	40 (43)	26 (42)	63 (52)	160 (51)	
Yes	301 (51)	53 (57)	36 (58)	58 (48)	154 (49)	
KPS, Median (Q1,Q3)	90 (80, 90)	90 (80, 90)	90 (80, 90)	90 (80, 90)	90 (80, 90)	0.176
Status, *n* (%)						<0.001
Alive	174 (29)	43 (46)	23 (37)	40 (33)	68 (22)	
Dead	416 (71)	50 (54)	39 (63)	81 (67)	246 (78)	
Overall survival time, Median (Q1,Q3)	24.17 (9.9, 50.99)	38.9 (12.87, 59.8)	18.05 (7.53, 50.99)	28.17 (11.9, 53.8)	20.4 (9.72, 48.59)	0.012
Air quality parameters						
AQI, Median (Q1, Q3)	89 (83, 102)	86 (81, 91)	87 (81, 96)	93 (85, 108)	90.5 (84, 104)	<0.001
Air stage, *n* (%)						0.049
Good	438 (74)	78 (84)	50 (81)	82 (68)	228 (73)	
Light pollution	95 (16)	11 (12)	10 (16)	21 (17)	53 (17)	
Median pollution	57 (10)	4 (4)	2 (3)	18 (15)	33 (11)	
PM2.5, Median (Q1, Q3)	61 (52, 76)	55 (47, 64)	58.5 (45.25, 71.75)	69 (55, 81)	62 (53, 77)	<0.001
PM10, Median (Q1,Q3)	82 (69, 97)	72 (66, 87)	75 (64, 89.75)	86 (72, 100)	82 (70, 97)	0.002
NO_2_, Median (Q1,Q3)	41 (25, 50)	38 (25, 46)	35.5 (22.25, 44)	43 (28, 62)	41 (28.25, 50)	0.007
SO_2_, Median (Q1,Q3)	27 (18, 51)	25 (17, 56)	35 (19, 55.75)	28 (20, 54)	24.5 (18, 47)	0.161
CO, Median (Q1,Q3)	0.91 (0.82, 1.05)	0.88 (0.8, 0.98)	0.89 (0.79, 0.98)	0.97 (0.85, 1.11)	0.91 (0.82, 1.06)	0.002
O3, Median (Q1,Q3)	110 (57, 125.75)	113 (85, 124)	114 (71.25, 130)	70 (52, 116)	111 (57, 129)	<0.001
Complications						
Hypertension, *n* (%)						0.037
No	366 (62)	49 (53)	47 (76)	76 (63)	194 (62)	
Yes	224 (38)	44 (47)	15 (24)	45 (37)	120 (38)	
Diabetes, n (%)						0.323
No	533 (90)	82 (88)	57 (92)	105 (87)	289 (92)	
Yes	57 (10)	11 (12)	5 (8)	16 (13)	25 (8)	
Hyperlipemia, *n* (%)						0.024
No	538 (91)	88 (95)	57 (92)	102 (84)	291 (93)	
Yes	52 (9)	5 (5)	5 (8)	19 (16)	23 (7)	
Heart. failure, n (%)						0.111
No	579 (98)	89 (96)	61 (98)	121 (100)	308 (98)	
Yes	11 (2)	4 (4)	1 (2)	0 (0)	6 (2)	
ACS, *n* (%)						0.813
No	575 (97)	90 (97)	60 (97)	119 (98)	306 (97)	
Yes	15 (3)	3 (3)	2 (3)	2 (2)	8 (3)	

Abbreviation: ACS, Acute Coronary Syndromes; AQI, air quality index; BMI, Body Mass Index; IQR, interquartile range; KPS, Karnofsky Performance Status.

**FIGURE 2:**
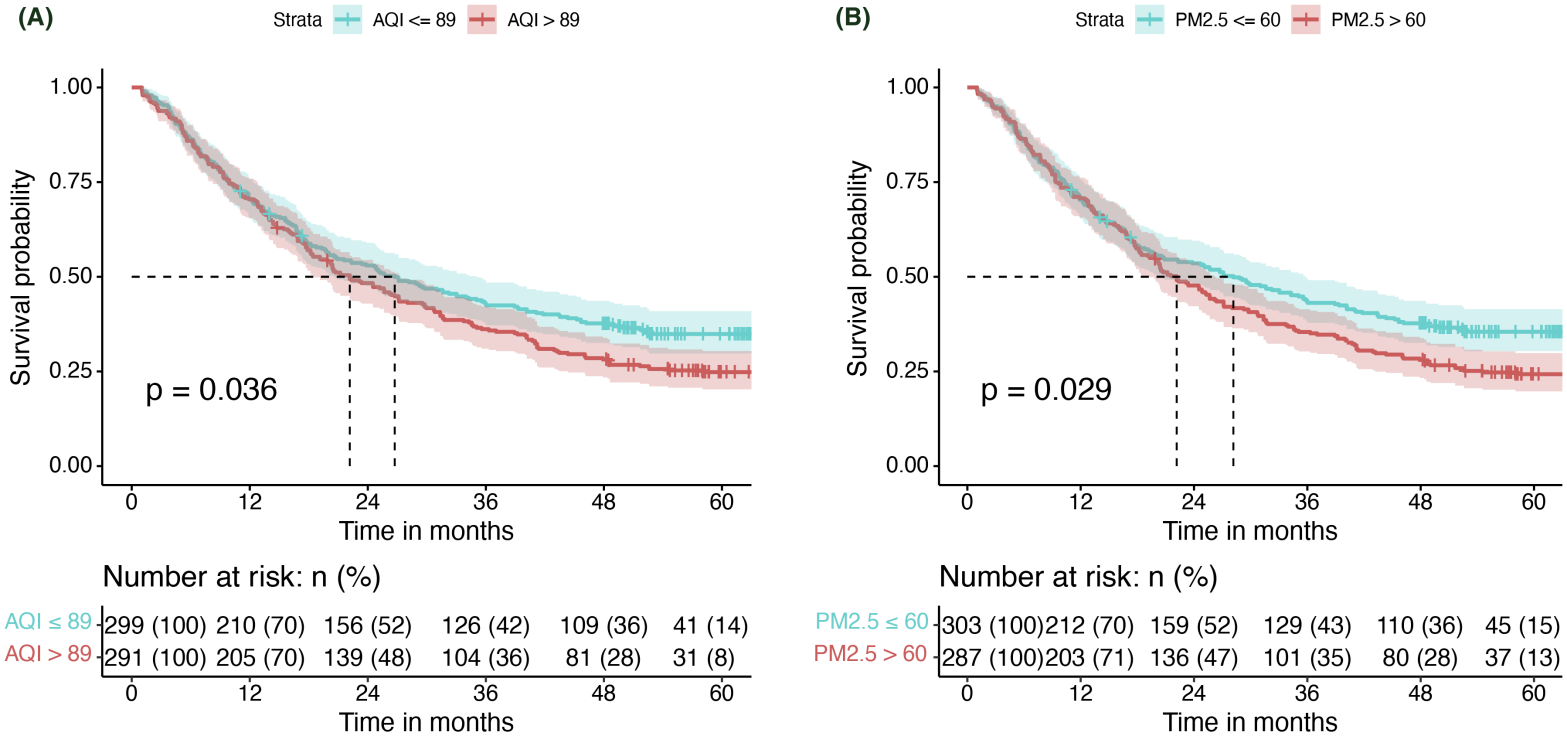
Kaplan–Meier curves for NSCLC patients with different air quality exposure. (A) Kaplan–Meier curves for OS in patients in the AQI≤89 and AQI >89 groups. (B) Kaplan–Meier curves for OS in different PM2.5 groups

**FIGURE 3:**
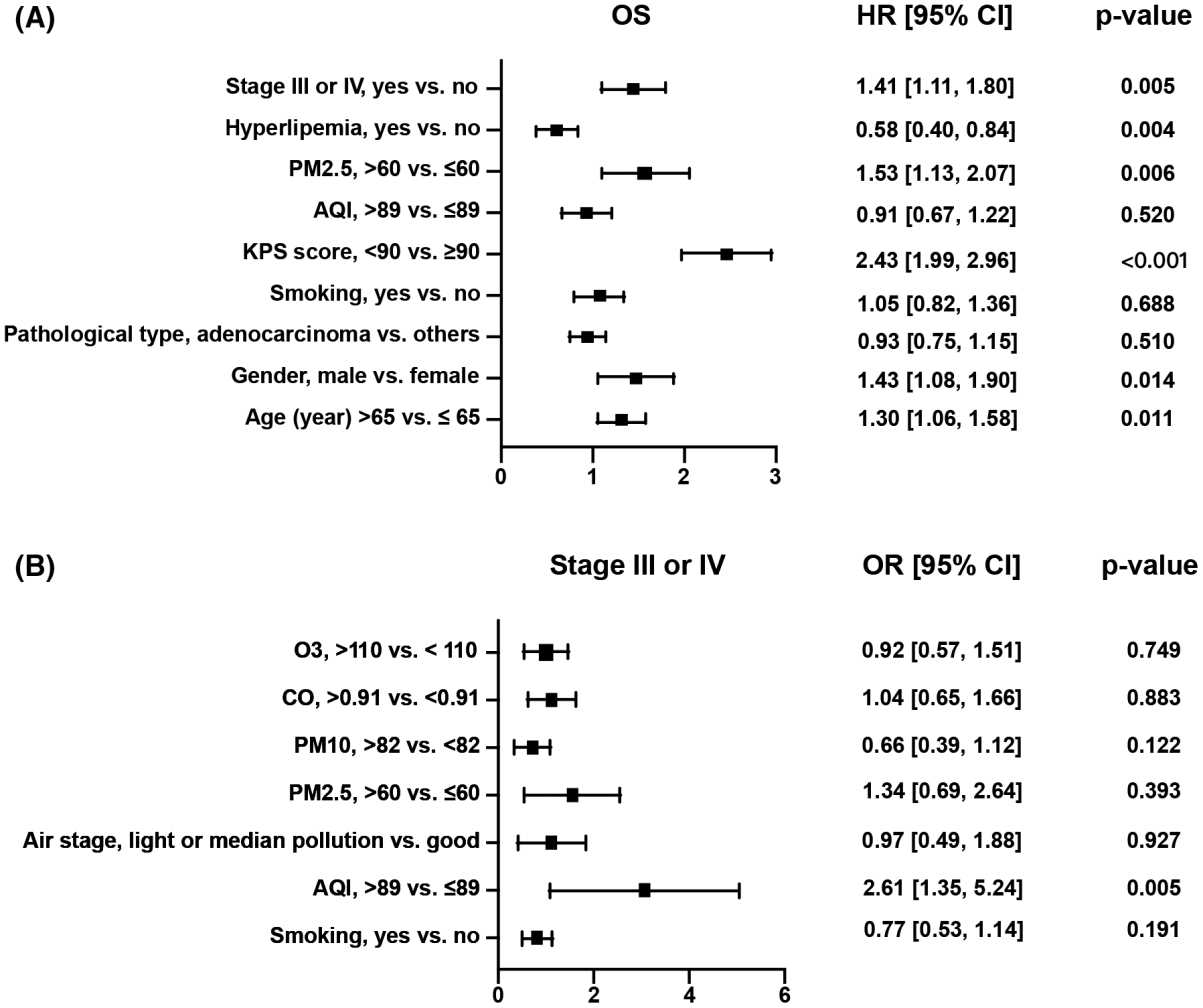
Forest plot for multifactorial analysis of different endpoints in patients with NSCLC. (A) Forest plot for multifactor analysis of the risk of overall mortality. (B) Forest plot for multifactor analysis of the risk of stage III or IV NSCLC

Kaplan–Meier curves showed that among NSCLC patients, those exposed to AQI > 89 in the 6 months prior to diagnosis had lower overall survival than controls (log‐rank, *p* = 0.036) (Figure 2A). Similarly, patients exposed to PM2.5 levels above 60 had lower overall survival than controls (log‐rank, *p* = 0.029) (Figure 2B). To explore the impact of air quality on tumor staging and long‐term survival in NSCLC patients, we performed univariate analysis of the above factors. The data showed that exposure to high levels of PM2.5 in NSCLC patients was a risk factor for overall patient survival (HR = 1.35, 95% CI 1.11–1.64, *p* = 0.002), whereas AQI (OR = 2.61, 95% CI 1.78–3.86, *p* < 0.001), air stage (OR = 1.91, 95% CI 1.22–3.09, *p* = 0.006), PM2.5 (OR = 2.32, 95% CI 1.60–3.41, *p* < 0.001), PM10 (OR = 1.45. 95% CI 1.01–2.11, *p* = 0.048), NO_2_ (OR = 1.48, 95% CI 1.02–2.15, *p* = 0.038), and elevated CO levels (OR = 1.47, 95% CI 1.02–2.14, *p* = 0.041) were risk factors for higher tumor stage (stage III or IV) in NSCLC patients. In contrast, elevated O_3_ levels were an effective protective factor for the above endpoint events (OR = 0.65, 95% CI 0.45–0.94, *p* = 0.021) (Table 2). Based on statistical results and clinical experience, we included factors with potential predictive value in the multifactorial analysis. The results showed that elevated PM2.5 levels (HR = 1.53, 95% CI 1.13–2.07, *p* = 0.006) were likewise an independent risk factor for long‐term survival (Figure 3A). In addition, elevated AQI levels (HR = 2.61, 95% CI 1.35–5.24, *p* = 0.005) were an independent risk factor for achieving higher tumor stage (stage III or IV) in NSCLC patients (Figure 3B).

**TABLE 2:** Univariate regression analysis on NSCLC patients for different endpoints

Variants	OS		Stage III or IV	
	HR	*p* value	OR	*p* value
Age (years), >65 versus ≤65	1.38 [1.14, 1.67]	0.001	0.92 [0.64, 1.34]	0.668
Gender, male versus female	1.52 [1.22, 1.89]	<0.001	0.84 [0.56, 1.26]	0.41
Pathological type, adenocarcinoma versus others	0.75 [0.61, 0.91]	0.004	1.27 [0.87, 1.84]	0.219
Smoking, yes versus no	1.32 [1.09, 1.60]	0.005	0.70 [0.49, 1.02]	0.064
KPS score, <90, versus >90	2.40 [1.97, 2.93]	<0.001	1.07 [0.74, 1.56]	0.707
AQI, >89 versus <89	1.23 [1.01, 1.49]	0.036	2.61 [1.78, 3.86]	<0.001
Air stage, light or median pollution versus good	1.07 [0.86, 1.33]	0.541	1.91 [1.22, 3.09]	0.006
PM2.5, > 60 versus < 60	1.35 [1.11, 1.64]	0.002	2.32 [1.60, 3.41]	<0.001
PM10, >82 versus <82	1.19 [0.98, 1.44]	0.082	1.45 [1.01, 2.11]	0.048
NO_2_, >41 versus <41	1.03 [0.85, 1.25]	0.782	1.48 [1.02, 2.15]	0.038
SO_2_, >27 versus <27	0.96 [0.79, 1.17]	0.707	0.96 [0.67, 1.39]	0.833
CO, >0.91 versus <0.91	1.02 [0.84, 1.24]	0.81	1.47 [1.02, 2.14]	0.041
O_3_, >110 versus <110	0.92 [0.76, 1.12]	0.403	0.65 [0.45, 0.94]	0.021
Hypertension, yes versus no	1.03 [0.84, 1.25]	0.788	0.99 [0.68, 1.46]	0.977
Diabetes, yes versus no	1.14 [0.83, 1.57]	0.427	0.90 [0.50, 1.71]	0.745
Hyperlipemia, yes versus no	0.63 [0.44, 0.91]	0.014	1.55 [0.79, 3.34]	0.23
Heart failure, yes versus no	1.25 [0.62, 2.53]	0.526	0.42 [0.12, 1.47]	0.156
ACS, yes versus no	1.18 [0.63, 2.22]	0.601	0.71 [0.25, 2.30]	0.531
Stage III or IV, yes versus no	1.39 [1.10, 1.75]	0.006	—	—

Abbreviation: ACS, Acute Coronary Syndromes; AQI, air quality index; BMI, Body Mass Index; IQR, interquartile range; KPS, Karnofsky Performance Status.

**FIGURE 4:**
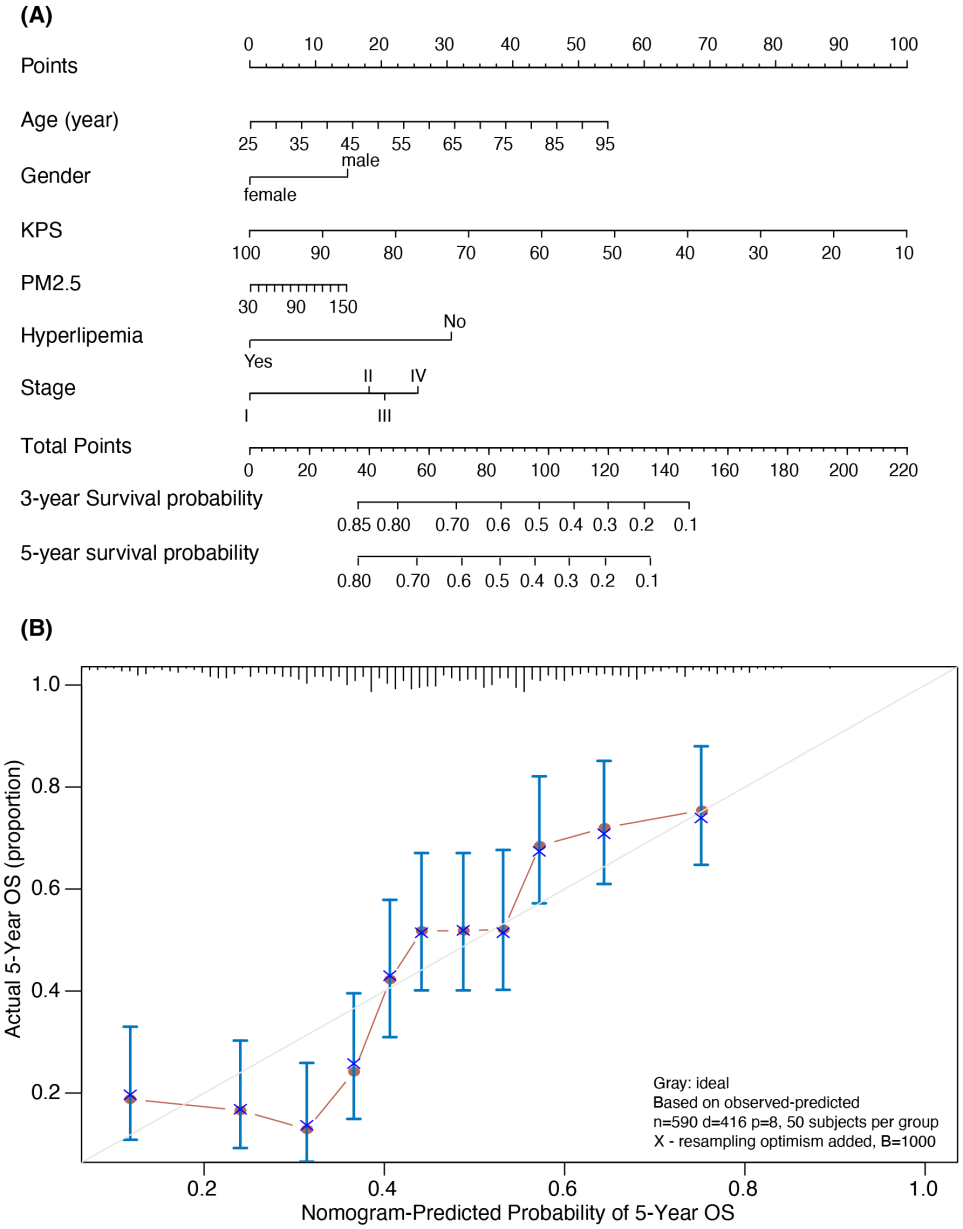
Nomogram of 5‐year survival estimates in patients with NSCLC and its predictive performance. (A) Nomogram of 5‐year survival estimates in patients with NSCLC. (B). Validation of the nomogram in estimating the predictive performance of patients with NSCLC (*n* = 590)

**FIGURE 5:**
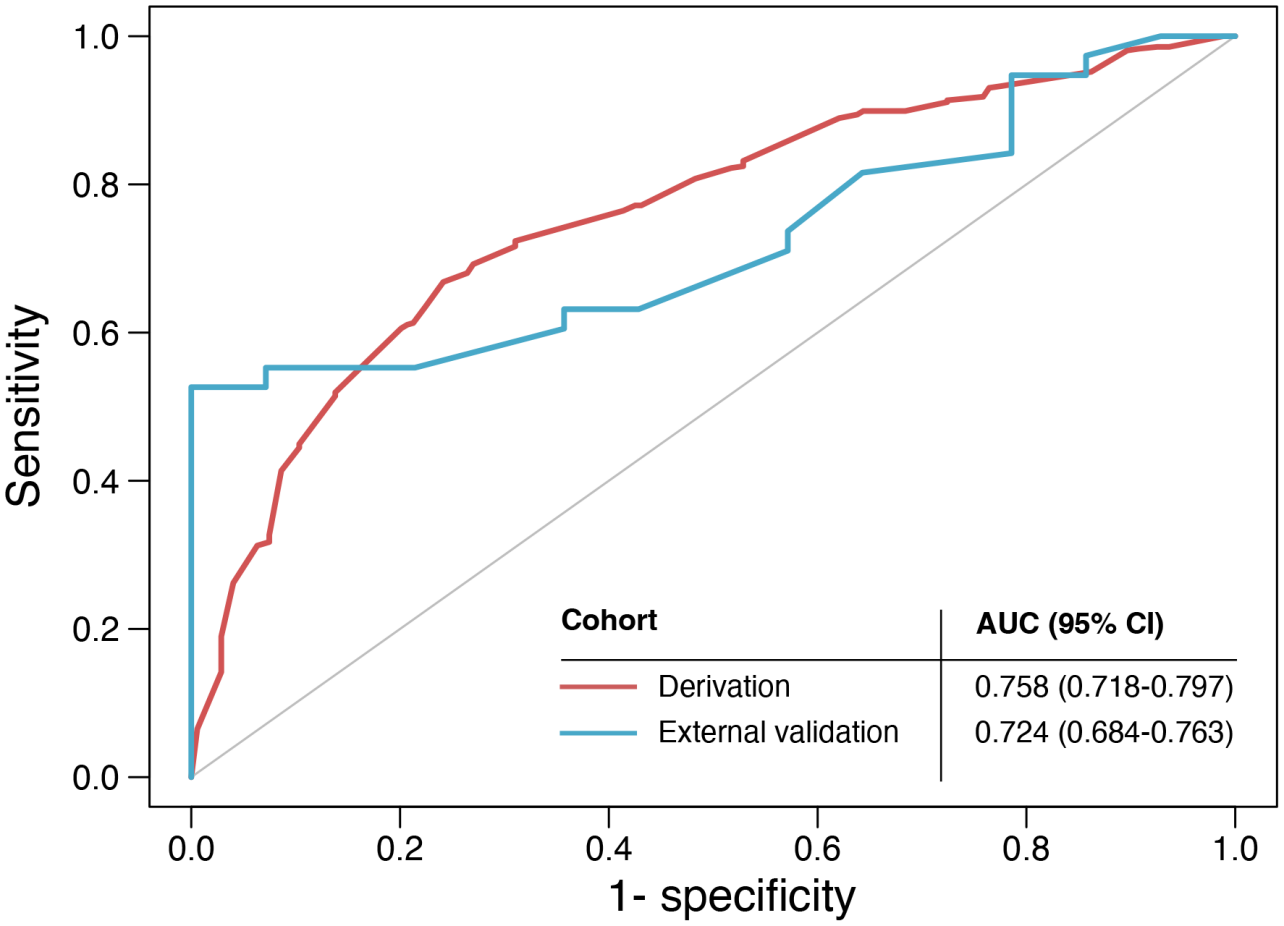
ROC curve for derivation and external validation

Overall survival at 3 and 5 years is an essential metric for assessing long‐term survival in patients with NSCLC. The successful prediction may help clinicians in the treatment selection of patients. Based on multivariate regression, we selected age, gender, KPS score, PM2.5, and the presence of hyperlipidemia to build prediction models. To quantify the contribution of each factor to the 5‐year overall survival, we generated a model displayed by nomogram as shown in Figure 4A. The calibration curve showed good calibration of the model (C‐statistic = 0.758) (Figure 4B). We collected 46 NSCLC patients from Sichuan Cancer Hospital in the external validation step. The ROC curve showed an AUC of 0.758 (0.718–0.797) for predicting 5‐year overall survival compared with an AUC of 0.724 (0.684–0.763) for the external validation data (Figure 5).

## 4 DISCUSSION

This cohort study demonstrated the value of air quality parameters, especially AQI and PM2.5, in reflecting long‐term survival and tumor progression in NSCLC patients. Elevated AQI as well as PM2.5 levels are detrimental to the long‐term prognosis of NSCLC patients. We also developed a prediction model for long‐term survival events in NSCLC patients based on PM2.5 levels to facilitate clinicians' decision‐making.

A meta‐analysis led by Hamra et al. showed that both PM2.5 and PM10 contributed to the development of lung cancer, with PM2.5 having a greater effect. The meta‐estimates of adenocarcinoma associated with PM2.5 and PM10 were 1.40 (95% CI: 1.07, 1.83) and 1.29 (95% CI: 1.02, 1.63), respectively.^23^ However, there are fewer studies related to the effects of these two air pollutants on OS. Our results show that PM2.5 has a greater prognostic impact on NSCLC compared with PM10.

Several studies have found a significant relationship between PM2.5 and lung disease.^24^ PM2.5 is an air pollutant of ≤2.5 μm in diameter that is characterized by large surface area, small particle size,^25^ and high toxin absorption capacity.^26^ These properties give PM2.5 the potential to invade the minor respiratory tracts including alveolar tissues. PM2.5 included metals, black carbon, sulfates, polycyclic aromatic hydrocarbons, nitrates, and vehicle exhaust particles.^27^ It has been shown that exposing mice to PM2.5 results in a greater tendency to deposit PM2.5 into lung problems than other metal pollutants.^28^ Many biological activities, including coagulation homeostasis, cardiopulmonary function, and cytokine formation, are altered by PM2.5.^29^ Several studies have shown that PM2.5 may trigger chronic obstructive pulmonary disease (COPD) and lung cancer by activating AMP‐activated protein kinase (AMPK) catalytic subunit α1 and related signaling pathways such as signal transducer and activator of transcription (STAT)‐1.^30^ A small number of studies have also shown that nuclear factor‐κB (NF‐κB), mitogen‐activated protein kinase (MAPK), vascular endothelial growth factor receptor (VEGF), and interleukin (IL)‐8 signaling are involved in PM2.5‐induced lung injury.^31^

Our study shows that PM2.5 is a risk factor for long‐term survival and high stage of lung cancer (stage III or stage IV) in NSCLC patients and is an independent risk factor for survival. These patients who are exposed to high levels of PM2.5 for a long time often suffer from lung inflammation. Examples include asthma and COPD. Therefore, we speculate that the predisposing effect of PM2.5 on lung cancer may be related to inflammatory development. The tumor microenvironment is essential for tumorigenesis and progression, especially in NSCLC.^32^ The production of cytokines, inflammatory cells, and angiogenesis has been recognized to be associated with tumor cell proliferation and tumor metastasis.^33^ Many transcription and inflammatory factors play a role in the NSCLC tumor microenvironment including NF‐κB, STAT‐3, interleukin‐6 and ‐1β, and tumor necrosis factor‐alpha (TNF‐α).^34^ Exposure to PM2.5 can increase the proliferative and mobility of H1299 and A549 cells, and MMP‐1 and IL‐1β may be responsible for the effects of PM2.5.^35^ The polarization of alveolar macrophages may play a role in tumor growth and angiogenesis through the secretion of VEGF and IL‐8.^36^ Previous studies have shown that PM2.5 can induce the release of various pro‐inflammatory cytokines, including TNF‐α, granulocyte‐macrophage colony‐stimulating factor (GM‐CSF), and IL‐6, from HBE cells and macrophages, leading to airway inflammation.^37^, ^38^, ^39^ Thus, PM2.5‐induced alterations in the tumor microenvironment may promote tumor growth and metastasis by triggering angiogenic and inflammatory responses. Therefore, for NSCLC patients, we need to provide them with a clean environment or use devices such as air purifiers to improve air quality. It will benefit the long‐term quality of survival of these patients. There are few studies on the correlation between SCLC and environmental pollution. We found that previous studies showed that radon gas was associated with the occurrence of SCLC. Krewski et al. analyzed a pooled study of seven case–control studies and found a correlation between residential radon exposure and lung cancer, with the strongest relationship with SCLC.^40^ In addition, a case–control study in New Jersey conducted by Wilcox et al. found no significant increase in lung cancer risk with increasing radon levels. However, radon exposure showed a stronger effect on SCLC in both men and women.^41^

This study remains a retrospective cohort study. The air quality of each patient's environment was not entirely consistent, which led to the limited evidence‐based nature of this study. Future multicenter clinical trials are needed to demonstrate the impact of air quality, especially AQI and PM2.5, on long‐term survival in NSCLC patients.

## 5 CONCLUSION

Our results show that PM2.5 and AQI levels affect disease progression and long‐term survival of NSCLC patients. An overall survival prediction model based on PM2.5 levels could help clinicians predict the mortality in NSCLC patients.

## CONFLICT OF INTEREST

The authors declare that there are no competing interests.

## AUTHORS' CONTRIBUTION

LJ and WXY conducted the analysis and drafted the manuscript. GL completed studies and analyzed data. QLY, XMQ, ZHC, and XL contributed to the manuscript writing and data analysis. PWY, YZY, and ZXL added to the collection and analysis of clinical data. ZDX and JJH contributed to the research design, data analysis, writing the manuscript, and supervision of the investigation.

## ETHICAL APPROVAL AND CONSENT TO PARTICIPATE

The Clinical Research Ethics Committee approved clinical data with the Affiliated Dushu Lake Hospital of Soochow University.

## CONSENT FOR PUBLICATION

We have got consent from all the authors for publication.

## Data Availability

Original data is available if necessary.
